# Orthognathic Surgery Patients (Maxillary Impaction and Setback plus Mandibular Advancement plus Genioplasty) Need More Intensive Care Unit (ICU) Admission after Surgery

**Published:** 2015-03

**Authors:** Hamidreza Eftekharian, Barbad Zamiri, Shamseddin Ahzan, Mohamad Talebi, Kamal Zarei

**Affiliations:** 1 Dept. of Craniomaxillofacial Surgery, School of Dentistry, Shiraz University of Medical Sciences, Shiraz, Iran;; 2 Undergraduate Student, Student Research Committee, School of Dentistry, Shiraz University of Medical Sciences, Shiraz, Iran;; 3 Undergraduate Student, Student Research Committee, School of Dentistry, Shiraz University of Medical Sciences, Shiraz, Iran;; 4 Postgraduate, Dept. of Craniomaxillofacial Surgery, School of Dentistry, Shiraz University of Medical Sciences, Shiraz, Iran;

**Keywords:** Bimaxillary Orthognathic Surgery, Intensive Care Unit (ICU), Maxillary Impaction, Estimated Blood Loss

## Abstract

**Statement of the Problem:** Due to shortage of ICU beds in hospitals, knowing what kind of orthognathic surgery patients more need ICU care after surgery would be important for surgeons and hospitals to prevent unnecessary ICU bed reservation.

**Purpose:** The aim of the present study was to determine what kinds of orthognathic surgery patients would benefit more from ICU care after surgery.

**Materials and Method:** 210 patients who were admitted to Chamran Hospital, Shiraz, for bimaxillary orthognathic surgery (2008-2013) were reviewed based on whether they had been admitted to ICU or maxillofacial surgery ward. Operation time, sex, intraoperative Estimated Blood Loss (EBL), postoperative complications, ICU admission, and unwanted complications resulting from staying in ICU were assessed.

**Results:** Of 210 patients undergoing bimaxillary orthognathic surgery, 59 patients (28.1%) were postoperatively admitted to the ICU and 151 in the maxillofacial ward (71.9%). There was not statistically significant difference in age and sex between the two groups (*p*> 0.05). The groups were significantly different in terms of operation time (*p*< 0.001). Blood loss For ICU admitted patients was 600.00±293.621mL and for those who were hospitalized in the ward was 350.00±298.397 mL. Statistically significant differences were found between the two groups (*p*< 0.001). Moreover, there was a direct linear correlation between operation time and intraoperative estimated blood loss and this relationship was statistically significant (r=0.42, *p*< 0.001). Patients with maxillary impaction and setback plus mandibular advancement plus genioplasty were among the most ICU admitted patients (44%), while these patients were only 20% of all patients who were admitted to the ward. As a final point, the result illustrated that patients who were admitted to the ICU experienced more complication such as bleeding, postoperative nausea, and pain (*p*< 0.001).

**Conclusion:** Orthognathic surgery patients (maxillary impaction and setback plus mandibular advancement plus genioplasty) due to more intraoperative bleeding and postoperative nausea and pain would benefit from ICU admission after surgery.

## Introduction

 Orthognathic surgery is used to correct facial anatomy in patients with dentofacial disorders as a part of frequently-performed hospital surgeries. This surgery results in a considerable blood loss to the extent that patients undergoing such operations sometimes need blood transfusions. The reason for such considerable blood loss is the high vascular nature of the maxillofacial area as well as the difficulty of access to the area under surgery for hemostasis purposes.[1] Currently, orthognathic surgeries are performed most frequently.[2] One of the most serious risks of bimaxillary orthognathic surgeries is severe blood loss during such operations.[3] Among the most important challenges that anesthesiologist and surgeons usually face are intraoperative bleeding during orthognathic surgery, the need for transfusion of blood products, postoperative pain, side effects such as nausea and vomiting, postoperative agitation and shivering, severe swelling due to airway manipulation and extent of surgery, and intermaxillary fixation. Therefore, some orthognathic patients need special hospital care for continuous monitoring of vital signs and nursing interventions. On the other hand, undesirable side effects of intensive care unit (ICU) admission such as resistant infections in the hospital, unknown fever, nosocomial pneumonia, long-term problems of keeping the endotracheal tube, overdosing the patient with sedating agents to tolerate ventilator system could jeopardize the patient's health.[4-5] 

 ICUs cater for patients with the most severe and life-threatening illnesses and injuries, which require constant close monitoring and support from specialist, equipment and medication in order to ensure normal bodily functions. They are staffed by highly trained doctors and critical care nurses who have been specialized in caring for seriously ill patients. The complexity of the surgery, the surgeon's experience and skill, the operation time, intraoperative blood loss, and the patient’s hemodynamics are indications that require ICU admission.[6] Measuring the quality of services provided in the ICU is very difficult because of the qualitative nature of such services, but the mortality rate in the ICU and the length of stay in the unit are two core indices for explanation of the offered services.[7]The ICU is one of the places where medical errors are likely to occur because of the complexity of patient care.[8] Furthermore, as the patients are heavily cared and simultaneously exposed to complex treatment interventions, they are highly susceptible to adverse effect. Although the possibility of human error in patient care in the ICU is high.[9-10] the cost of stay in ICU is a matter of great concern.[11-12] The ICU incurs a significant cost that accounts for 8 to 20 percent of total hospital costs. Treatment of patients before their admission to ICU would possibly change the relation between the patient's physiological scoring and the patient outcomes.[7-8,13-14] Accordingly, the present study was performed on patients with corrective orthognathic surgery who referred to Maxillofacial Surgery Department of Chamran Hospital (Shiraz, Iran) during the last five years. This study examined the advantages, disadvantages, and problems of admission of orthognathic surgery patients to the ICU regarding the length of stay, age, sex, operation time, as well as anesthesia, post-operative complications, and intraoperative bleeding in comparison with patients who were not admitted to the ICU. Unfortunately, there are few articles about this issue. ICU admission is done based on the anesthesiologist and surgeon’s opinions. The aim of this study was to investigate which of the orthognathic surgery patients need more ICU care after surgery. Due to shortage of ICU beds in hospitals, this knowledge would be important for surgeons and hospitals to prevent unnecessary ICU bed reservation. 

## Materials and Method

 In this retrospective study, 210 patients undergoing bimaxillary orthognathic surgery (2008-2013) were reviewed based on the permission obtained through correspondence with authorities of Chamran Hospital. These records were searched and received from the hospital documents using the computer system in the hospital. Then the data related to orthognathic patients were collected by answering the questionnaires containing the patients’ personal information, operation time from beginning of incision until suturing, intraoperative estimated blood loss (EBL) based on the volume of suction bottle and the number of surgical gauze used during the operation , postoperative complications such as postoperative bleeding, nausea and vomiting, and postoperative pain measured through behavioral pain scale which is used in critically ill, sedated, and mechanically ventilated patients,[31] admission to the ICU according to the ICU admission standards specially patients who require postoperative hemodynamic monitoring/ ventilator support or extensive nursing care,[15] unwanted complications resulting from staying in the ICU and the type of operation. Since the expenses depend on various factors including discounts and deductions, it was excluded from the questionnaire. Then the collected data were codified, entered in SPSS (Version 18), and analyzed using statistical measures such as chi-square test to examine the impact of gender on admission to the ICU. Independent student’s T-test was performed to examine the possible effects of age, blood transfusion, and operation time. In addition, Mann-Whitney test was used to evaluate the effect of blood loss on staying or not staying in the ICU. Kendall's correlation coefficient was adopted to examine the relationship of age and operation time with the amount of blood loss during surgery. 

## Results

 In this study, 210 patients who had undergone bimaxillary orthognathic surgery were studied. Out of these participants, 74 (35.2%) were males and 136 (64.8%) were females. In addition, 151 patients (71.9%) were postoperatively admitted to the maxillofacial surgery ward and 59 (28.1%) were admitted to the ICU. Types of bimaxillary surgery for ICU admitted patients were as following: 26 patients (44%) had maxillary impaction and setback plus mandibular advancement plus genioplasty, 20 patients (34%) had maxillary setback and mandibular advancement, 13 patients (22%) had maxillary advancement and mandibular setback. Patients with maxillary impaction and setback plus mandibular advancement plus genioplasty were among the most ICU admitted patients (44%), while these patients constituted only 20% of the patients who were admitted to the maxillofacial ward. The Mean±SD age of the patients was 24.03±7.57 years with the minimum and maximum age of 12 and 57 years, respectively. The average length of stay in the ICU was 1.02±0.6 days and in the maxillofacial surgery ward, it was 5.33±2.19 days. Operation time lasted averagely for 4.35±1.21 hours and the estimated blood loss during operation was 458.19±315.43 mL. In addition, these patients received 286.43±243.27 mL of blood transfusion. The postoperative and intraoperative complications of the patients were as represented in Table 1. 

**Table 1:** Frequency of postoperative complications among the patients undergone bimaxillary orthognathic surgery admitted to the ICU in Chamran Hospital (1387-1392)

**Post operative complications**	**Frequency**	**Percent**
No complication	17	8.1
Pain	25	11.9
Vomiting	2	1.0
Pain and vomiting	116	55.2
Pain and vomiting and bleeding	50	23.8
Total	210	100

 The results of the study suggested that gender had no effect on being admitted to the ICU or maxillofacial ward (*p*> 0.05) (Table 2). The Mean±SD age of patients who were hospitalized in the maxillofacial ward was 24.08±7.3 years, while this number was 23.92±8.31 for those who were admitted to the ICU, but the observed difference was not statistically significant (*p*> 0.05). According to the results of the study, the average time of surgery for patients hospitalized in the ICU was 4.96±0.96 hours, and for the patients hospitalized in the maxillofacial ward, it was 4.11±1.21 hours, although the observed difference between the two groups was not significant (*p*> 0.05). 

**Table 2:** Relationship between sex and ICU admission in Chamran hospital, Shiraz, Iran (1387-1392)

		**ICU admission**		**Total**
		**Yes**	**No**	
SEX	Male	50	24	74
	Female	101	35	136
Total		151	59	210

 The findings revealed that patients in the ICU had lost more blood than the patient hospitalized in the maxillofacial ward, and the difference in blood loss between the two groups was statistically significant (*p*< 0.001) (Table 3). 

**Table 3:** Average intraoperative blood loss among the patients undergone bimaxillary orthognathic surgery admitted to the general ward and ICU in Chamran hospital, Shiraz, Iran (1387-1392)

**ICU** **admission**	**Mean**	**Std.** **Deviation**	**Median**	**Minimum**	**Maximum**
General ward	390.79	298.397	350.00	0	1300
ICU	630.68	293.621	600.00	0	1400
Total	458.19	315.453	400.00	0	1400

Table 4 shows the estimated blood loss during the surgery for male and female patients; there was no significant relationship between gender and the amount of blood loss during the surgery (*p*> 0.05). 

**Table 4:** Intraoperative blood loss for males and females undergone bimaxillary orthognathic surgery in Chamran hospital, Shiraz, Iran (1387-1392)

**Sex**	**Mean**	**Std.** **Deviation**	**Median**	**Minimum**	**Maximum**
1	490.68	328.476	475.00	0	1400
2	440.51	307.928	400.00	0	1400
Total	458.19	315.453	400.00	0	1400

 The results showed that the ICU admitted patients suffered more from bleeding, nausea, vomiting, and pain than those who were hospitalized in the maxillofacial ward (*p*< 0.001) (Table 5). 

**Table 5:** Frequency of postoperative complications among patients underwent bimaxillary orthognathic surgery admitted to maxillofacial ward in Chamran hospital, Shiraz, Iran (1387-1392)

**Postoperative complications**	**No.**	**Mean**	**Std. Deviation**
No complication	17	.00	.000
Pain	25	.56	1.083
pain and vomit	118	.27	.712
pain and vomit and bleeding	50	1.62	1.123
Total	210	.60	1.02

**Figure 1:**
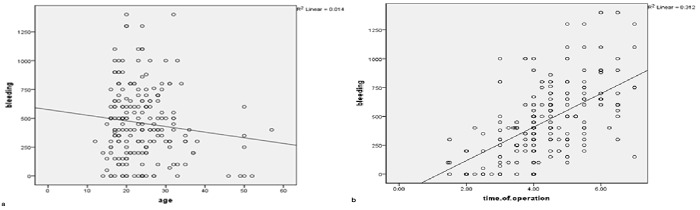
: Relationship between age and estimated blood loss during bimaxillary orthognathic surgery in Chamran hospital, Shiraz, Iran (1387-1392)  b: Relationship between operation time and estimated blood loss during bimaxillary orthognathic surgery in Chamran hospital, Shiraz, Iran (1387-1392)

 As displayed in Figure 1a, there existed a negative relationship between age and the amount of blood loss during the surgery (r=-0.033), however, this relationship was not statistically significant (*p*> 0.05). It was also shown that there is a direct linear relationship between operation time and the estimated blood loss during surgery (r= 0.42), and this relationship was statistically significant (*p*< 0.001) (Figure 1b). 

## Discussion

 Orthognathic surgery is a surgical correction for not only the abnormal shape of mandible, maxilla or both of them but also for the relations between mandible and maxilla.[16] Like many other surgeries, this type of surgery is coupled with some complications such as inflammation, infection, nausea, pain, and bleeding.[17] Admitting patients to the ICU immediately after the surgery is useful especially for patients who have had head and neck operations, since it provides a close monitoring of cardiovascular and respiratory system.[18] A cohort study performed by Lucienne *et al.*[19] on 401 patients admitted to the ICU showed a strong relationship between the admission time to the ICU and the survival rate. They reported that an hour delay in sending the patient to the ICU would increase the mortality rate and early access to the ICU would greatly benefit the critically-ill patients. However, the authors did not mention the reasons why the patients were admitted to the ICU. 

In our study, age and gender were examined as interfering factors. The effects of these factors were not significant on the patients being admitted to ICU or the general ward. However, it was noted that as the age increased, the amount of bleeding reduced among patients. Patients admitted to ICU had more blood loss during the operation than those hospitalized in general ward. Moreover, duration of the operation was longer for them, which was statistically in a direct relation with blood loss. The patients admitted to ICU showed more complications in the duration of their hospitalization.

 In a study by Panula *et al.*,[20] the most serious complication of orthognathic surgery was intraoperative estimated blood loss, especially in patients with a short neck and low ramus height. The reason was reported to be the difficulty of medical instrumentation during osteotomy which increased the risk of rupture of the internal maxillary artery.[20] Pineiro *et al.*[2] reported the average bleeding during orthognathic surgery to be 436.11 ±207.89mL. In our study, the Mean±SD amount of bleeding among the patients in ICU was 630.68± 293.621 mL,which was obviously higher than the average bleeding reported by Pineiro *et al.*; it was also higher than the intraoperative bleeding among patients admitted to general ward with a value of 390.79±298.397 mL. This suggests that according to ICU admission standards (diagnostic model, class G), patients with excessive bleeding require hemodynamic monitoring and thus admission to the ICU.[15] 

 Piñeiro-Aguilar *et al.*[2] found a positive relationship between the length of surgery (including anesthesia time) and the need for patient care so that all patients whose operations lasted more than 4 hours and 28 minutes, needed special care. In this study, the average surgery duration for patients hospitalized in ICU was 4 hours and 37 minutes that shows the patients’ need for intensive care. Meanwhile, the average operation time for patients admitted to the general ward was 4 hours and 6 minutes that was significantly lower than the time for patients admitted to ICU. Also, in a study by Grag *et al.*[22] on 411 patients in a hospital in UK, it was found that the overall mean operation time for bimaxillary osteotomy, bilateral sagittal split osteotomy, and LeFort 1 osteotomy was 4 hours and 30 minutes. However, the relationship between the operation time and blood loss was not examined in their study. 

 In a study by Pineiro *et al.*,[2]it was noted that bimaxillary surgery resulted in a major volume of blood loss which was directly related to the operation time and the magnitude of interventions. The results indicated a linear relationship between the operation time and intraoperative bleeding in such a way that longer operation time would result in more bleeding, and thus in higher morbidity rates and the need for more intensive care. Similarly, it was found that both the intensity of anesthesia and operation time was linearly correlated with morbidity after surgery, postoperative outcomes, and the need for hospitalization following the operation.[17, 23] 

 The results of the study indicated that patients who were admitted to the ICU experienced more pain, nausea, and bleeding than those in the general ward. According to Azzam *et al.*[23] pain is common in the ICU. Besides, one of the complications of orthognathic surgery is pain.[17] 

 A retrospective study by Silva *et al.*[17] on 553 patients who underwent orthognathic surgery in California showed that the incidence of nausea and vomiting in patients after surgery and during the hospitalization is high with a rate of 40.08%. However, similar to the present, nausea in patients after discharge from hospital was not studied. 

 Silva *et al.* reported some risk factors for postoperative nausea and vomiting among the patients undergoing orthognathic operations. These risk factors include having young ages (15-25 years), surgery operations lasting more than an hour, surgery on maxilla, and presence of high level of pain and consequent receiving opioid medication after the operation. 

 The results of the studies indicated that an increase in the operation time would raise the risk of nausea and vomiting. Sinclair *et al.*[24] found that the incidence of postoperative nausea and vomiting from 2.8% in patients with operation time <30 minutes increased to 27.7% among patients with the operation time of 150-180 minutes.[25] The reason was probably the use of emetic drugs specially anesthetics.[26] As shown in the current study, patients who were admitted to the ICU had a longer operation time with increased chances of nausea and vomiting. 

 Patients with postoperative nausea and vomiting (PONV) usually noted substantial relief of their emetic symptoms after expulsion of their gastric contents, especially those who vomited blood clots. However, the main role of blood in the stomach, as an important emetic factor, is not well understood.[17] This may be more salient among patients admitted to the ICU due to their extensive bleeding. Recent studies suggest that nausea and vomiting are two different biological phenomena that must be studied separately.[27] However, in this study and the one by Silva *et al*.[17] both nausea and vomiting were studied together. 

 Bleeding in the ICU is a normal phenomenon that is not only limited to patients with postoperative trauma.[28] In a study on the frequency and severity of bleeding in medical-surgical ICU, it was noted that 90% of the patients experienced bleeding because of 480 different events. Besides, 20% of the patients had severe bleeding that lasts more than 4 days. Only 15% of bleeding cases were observed in surgical site while 38% were found in the catheter entry into the vein, 38% at the endotracheal tube site, and only 16% of bleedings had a gastrointestinal source; these reasons account for more than 50% of the causes of bleeding.[29] 

 In another study, it was noted that coagulation abnormalities and stress-induced mucosal lesions are two of the most prevalent risk factors for significant bleeding in ICU patients.[28] In the ICU department, occult or visible bleeding may develop as a result of physiological stress caused by clinical interventions or as a result of the stress and intervention itself. Mechanical ventilation for more than 48 hours and coagulopathy are the two major risk factors for stress induced bleeding in the upper part of the gastrointestinal tract.[30] 

We believe that more extensive studies are needed on ICU admission of patients who have undergone bimaxillary orthognathic surgery. There must also be a thorough examination on other aspects such as the patients’ jaw movements, especially when performing mandibular setback or mandibular advancement as well as their effect on some specific patients’ air way, and the patient’s condition before the surgery which may have some effects on admitting patients to the ICU or the general ward.

The findings also show that more experienced nurses at the ICU have an effective documentation experience (beneficial for maintenance of the treatment) compared to the initial documentation done by the less experienced nurses at the general wards.

## Conclusion

Orthognathic surgery patients (maxillary impaction and setback plus mandibular advancement plus genioplasty) who experience more intraoperative bleeding, postoperative nausea, and pain would benefit from ICU admission after the surgery.

## Notes

**Conflicting Interest: **The authors declare no conflict of interest.
